# Design and fabrication of a Nano-based neutron shield for fast neutrons from medical linear accelerators in radiation therapy

**DOI:** 10.1186/s13014-020-01551-1

**Published:** 2020-05-11

**Authors:** Younes Afkham, Asghar Mesbahi, Abdolali Alemi, Farhad Zolfagharpour, Nasrollah Jabbari

**Affiliations:** 1 Department of Medical Physics, Faculty of Medicine, Urmia University of Medical Sciences, Urmia, Iran; 2 Molecular Medicine Research Center, Tabriz University of Medical Sciences, Tabriz, Iran; 3 Department of Inorganic Chemistry, Faculty of chemistry, Tabriz University, Tabriz, Iran; 4 Department of Physics, Faculty of Basic Sciences, University Of Mohaghegh Ardebili, Ardabil, Iran; 5 Solid Tumor Research Center, Cellular and Molecular Medicine Institute, Urmia University of Medical Sciences, Urmia, Iran

**Keywords:** Nanoparticle, Fe_3_o_4_, B_4_c, Silicone resin, Nanoshield, Neutron, Shielding, Photo-neutron

## Abstract

**Background:**

Photo-neutrons are produced at the head of the medical linear accelerators (linac) by the interaction of high-energy photons, and patients receive a whole-body-absorbed dose from these neutrons. The current study aimed to find an efficient shielding material for fast neutrons.

**Methods:**

Nanoparticles (NPs) of Fe_3_O_4_ and B_4_C were applied in a matrix of silicone resin to design a proper shield against fast neutrons produced by the 18 MeV photon beam of a Varian 2100 C/D linac. Neutron macroscopic cross-sections for three types of samples were calculated by the Monte Carlo (MC) method and experimentally measured for neutrons of an Am-Be source. The designed shields in different concentrations were tested by MCNPX MC code, and the proper concentration was chosen for the experimental test. A shield was designed with two layers, including nano-iron oxide and a layer of nano-boron carbide for eliminating fast neutrons.

**Results:**

MC simulation results with uncertainty less than 1% showed that for discrete energies and 50% nanomaterial concentration, the macroscopic cross-sections for iron oxide and boron carbide at the energy of 1 MeV were 0.36 cm^− 1^ and 0.32 cm^− 1^, respectively. For 30% nanomaterial concentration, the calculated macroscopic cross-sections for iron oxide and boron carbide shields for Am-Be spectrum equaled 0.12 cm^− 1^ and 0.15 cm^− 1^, respectively, while they are 0.15 cm^− 1^ and 0.18 cm^− 1^ for the linac spectrum. In the experiment with the Am-Be spectrum, the macroscopic cross-sections for 30% nanomaterial concentration were 0.17 ± 0.01 cm^− 1^ for iron oxide and 0.21 ± 0.02 cm^− 1^ for boron carbide. The measured transmission factors for 30% nanomaterial concentration with the Am-Be spectrum were 0.71 ± 0.01, 0.66 ± 0.02, and 0.62 ± 0.01 for the iron oxide, boron carbide, and double-layer shields, respectively. In addition, these values were 0.74, 0.69, and 0.67, respectively, for MC simulation for the linac spectrum at the same concentration and thickness of 2 cm.

**Conclusion:**

Results achieved from MC simulation and experimental tests were in a satisfactory agreement. The difference between MC and measurements was in the range of 10%. Our results demonstrated that the designed double-layer shield has a superior macroscopic cross-section compared with two single-layer nanoshields and more efficiently eliminates fast photo-neutrons.

## Introduction

Application of external photon beam radiation therapy with the energy greater than 10 MeV produces unwanted photo-neutrons. These neutrons are produced at the head of the medical linear accelerator (linac) through photon interaction with the nuclei of materials with a high atomic number, found in target, primary, and secondary collimators, flattening filter, etc. [1–3]. Consequently, neutron-absorbing materials have been utilized as barriers in maze and treatment room walls, examples of which include concrete and borated polyethylene in the doors of treatment rooms [4–6]. These barriers must be of sufficient thickness to protect radiation workers in radiation therapy installations [7].

In radiotherapy with megavoltage photon beams the risk of secondary primary cancer increases in out-of-field organs due to the stray dose caused by the photons scattered from the treatment head, photons leakage through treatment head components, photo-neutrons produced in the treatment head, and photons scattered within the patient [8–11]. The fractional radiation dose contribution of photo-neutrons relative to scattered photons in out-of-field is about 28% [12].

Photo-neutrons are particles with higher penetration and relative biological effectiveness (RBE) with respect to charged particles and sparsely ionizing radiations respectively [13, 14]. As a consequence, the radiation quality factor (QF) for photo-neutrons produced in radiation therapy with the energy range of 0.1–2 MeV is 20 [13, 14]. Therefore, in regard to the cancer induction, even small doses of photo-neutrons outside of the target volume can be important [8, 15].

Several studies have been conducted on the application of novel shielding materials for neutrons [16–18], most of which aimed to shield thermal neutrons to prevent these neutrons from emitting out of treatment rooms [19–21]. In recent studies, new composites have been applied for neutron shielding, and various particle sizes have been utilized as filters in different base materials for thermal neutron shielding, including micro- and nano-sized boron carbide and dense polyethylene [5, 7, 22]. Compared with micro-sized particles, nano-sized particles can be more homogeneously spread in the shield matrix with less aggregation. Hence, nanocomposite shields present higher shielding capabilities in radiation protection against both thermal and fast neutrons [21–23]. In addition, shielding materials with nanoparticles (NPs) shows high levels of tensile and flexural strengths [21–23]. The size and concentration of NPs are two factors influencing the fabrication of neutron shields [21–23]. The Monte Carlo (MC) simulation method plays a vital role in understanding the effect of parameters such as particle size, concentration, and radiation energy. Also, in designing a new shield, it provides the possibility of selecting various materials in different combinations.

Concrete and boron carbide-based products are commonly employed as shields for neutrons in radiation therapy due to their properties such as low price and high absorbing cross-sections for shielding neutrons [1, 22, 24]. Photo-neutrons are produced at the head of medical linac by the interaction of high-energy photons (E > 10 MeV), and patients receive a whole-body-absorbed dose from these neutrons [2, 3, 14]. To the best of our knowledge, no study has been dedicated to shielding materials with flexibility and low weight to prevent these neutrons from reaching patients.

According to several studies, composites containing nano-boron such as boron carbide, lead borate, and bismuth borate have been used for thermal neutron attenuation due to the high cross-section of boron as a neutron absorber. Iron oxide has shown high efficiency in attenuating and eliminating fast neutrons [17, 25, 26]. In this study, a double-layer shield consisting of nano-iron oxide and nano-boron carbide was designed to attenuate photo-neutrons with a broad energy range, which is the novelty of the present study.

Reviewing the published literature revealed the unavailability of a shield for protecting patients from fast generated neutrons in linacs. Therefore, the present investigation aimed to find an efficient shielding material for fast neutrons by applying nano-boron carbide and nano-iron oxide in a base of silicone resin. The goal of this research was to evaluate and compare the attenuating characteristics of both nanomaterials (boron carbide and iron oxide) for the range of fast neutron energies produced in radiation therapy and then design and fabricate a shield with a reasonable yield of neutron attenuation.

## Methods

### Shielding parameters

#### Macroscopic cross-section

This parameter, denoted by Σ, describes the probability of interaction of a neutron with a target material with units of cm^− 1^

1 $$ {\mathrm{I}}_{\mathrm{x}}={\mathrm{I}}_0{\mathrm{e}}^{-\Sigma \mathrm{x}}\mathrm{or}\kern0.5em \ln \left(\frac{\mathrm{I}\mathrm{x}}{10}\right)=-\Sigma \mathrm{x}\kern0.5em \mathrm{or}\kern0.5em \hbox{-} \Sigma =\kern0.5em \mathrm{l}/\mathrm{x}.\ln \left({\mathrm{I}}_{\mathrm{x}}/{\mathrm{I}}_0\right) $$

where I_0_ and I show the intensities of initial and attenuated neutrons, respectively, and X denotes the thickness of the absorber. Ix/I_0_ is called the transmission factor [27].

#### Neutron characterization

We evaluated the fluence and spectra of neutrons with energy ranging from 0.4 eV to 15 MeV, which is more than the energy of thermal neutrons (0.025 eV) [6]. For this reason, in the investigated spectra, all neutron energy ranges were used and considered as fast neutrons.

### Monte Carlo simulation

**Fig. 1:**
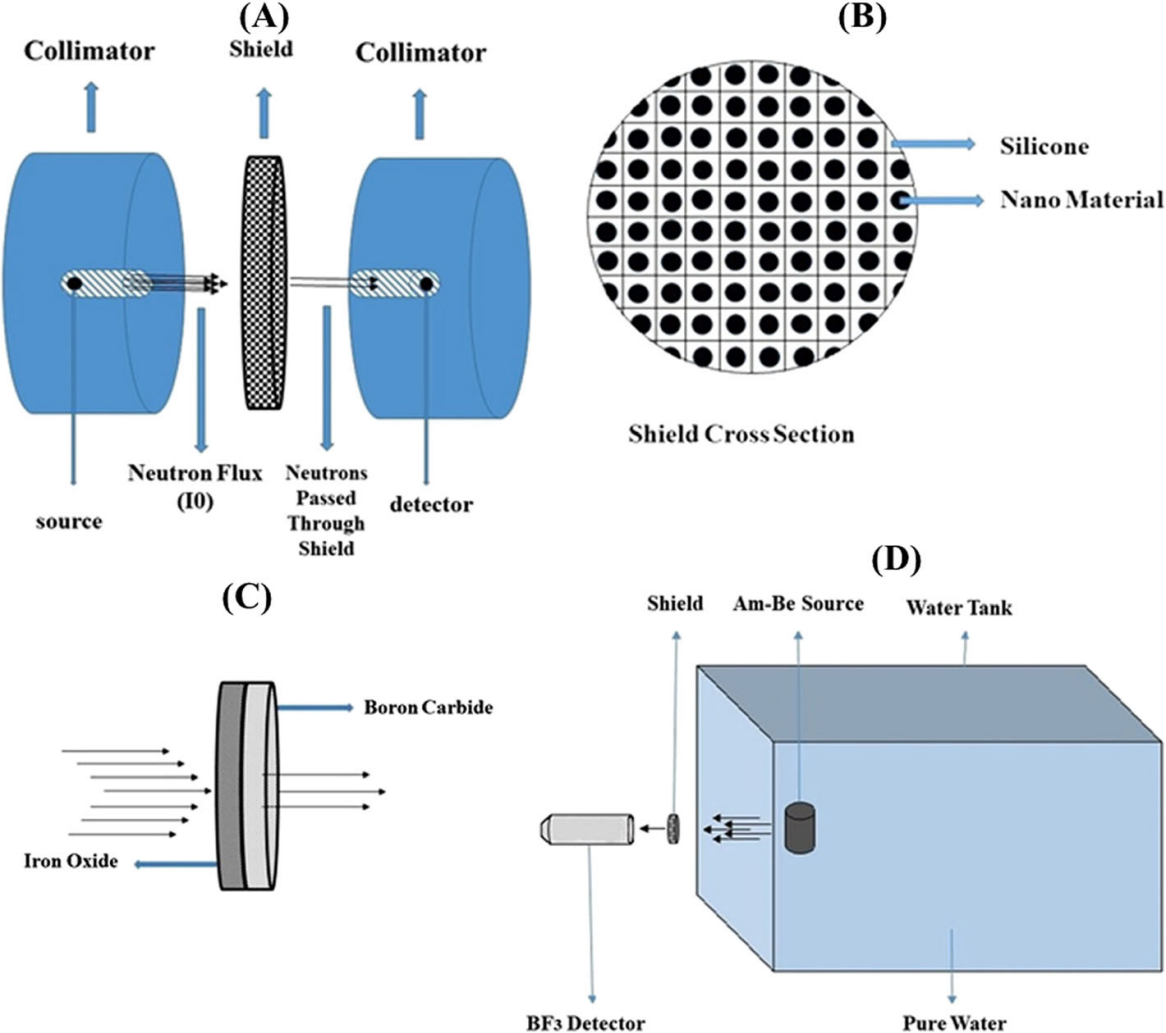
Simulated geometry and shield in MCNPX code: simulated geometry of environment (**a**), simulated lattice for shields (**b)**, joined configuration of B_4_C and Fe3O4 (**c**), and geometry of experimental setup (**d**)

In this study, simulations were performed using MCNPX MC code version 2.6.0 [28]. A narrow-beam geometry was modeled that included a point source (emitting neutrons vertically to the entrance surface of a detector), a collimator made of cadmium surrounding the point source, the designed nano-material shield, a sphere volume with diameter of 1 cm defined as a detector, and a collimator made of cadmium surrounding the detector (Fig. 1a). A shield was simulated between the source and the detector (Fig. 1a). Tally F4 (F4:n) was utilized for detecting the number of neutrons reaching the detector in terms of neutron/cm^2^. In MCNP MC code, the F4:n tally calculates the average neutron fluence per simulated source-neutron within the cell detector.

Initially, the fluence of neutrons in the detector cell (I_0_) was calculated for the narrow beam geometry without the nanoshields between the source and detector. Next, nanoshields with different thicknesses were placed between the source and detector cell, and the fluences of neutrons were calculated in the detector cell (I_x_) for each of them. Afterward, the transmission factors were determined by dividing I_x_ to I_0_. Finally, the macroscopic cross-section of each nanoshield in different concentrations and thicknesses was calculated by Eq. 1.

**Fig. 2:**
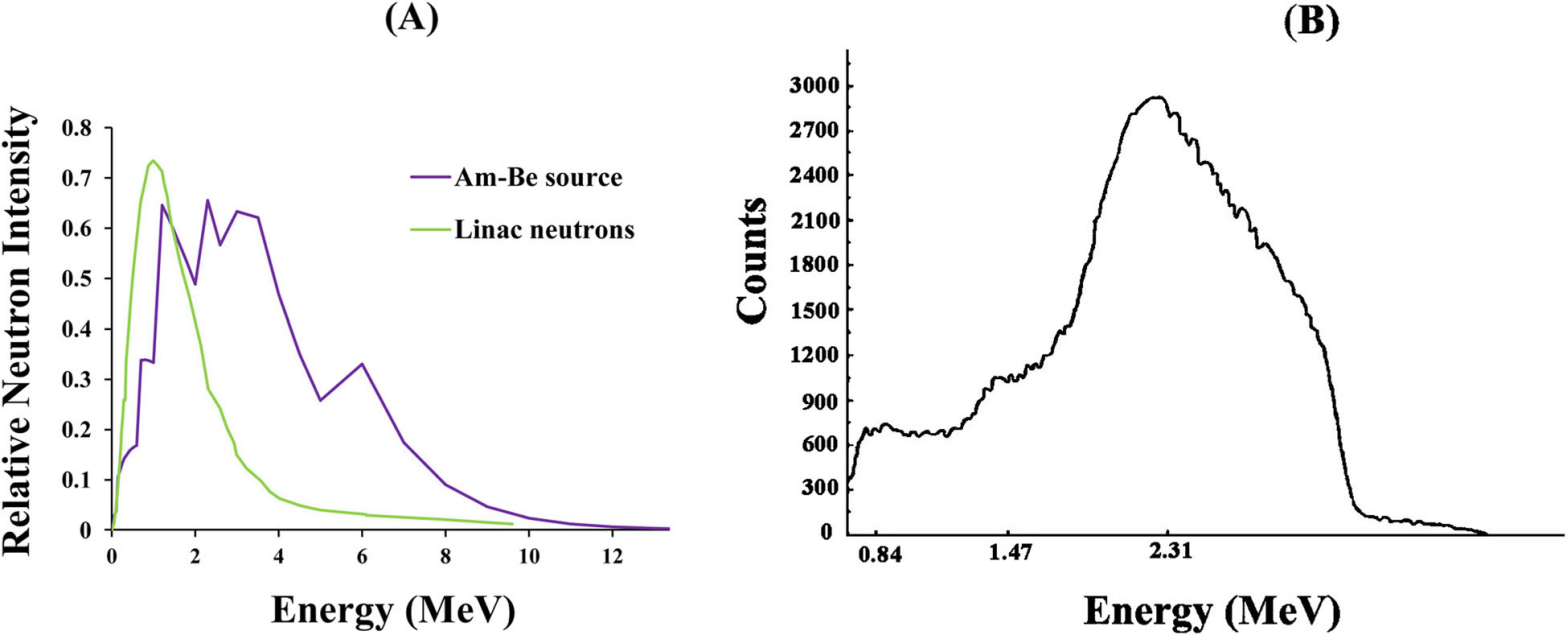
Neutron energy spectra: produced in linac head and Am-Be source (**a**), produced in Am-Be source in the laboratory (**b**)

Three types of neutron energies were used in our simulations. First, monoenergetic neutrons including 1–10 MeV with steps of 1 MeV were defined in our calculations. The second neutron source was the neutron spectra of an Am-Be source (Fig. 2a) obtained from previous studies [29, 30]. The third neutron source was the 18 MV photon beam of Varian 2100 C/D medical linear accelerator (Fig. 2a) obtained from other studies [3, 31, 32]. Finally, to compare our MC results with experimental measurements and to test the designed and fabricated nanoshields, the experimental measurements were performed with a neutron source of an Am-Be in the laboratory (Fig. 2b). Because of the lack of access to energy spectrum raw data from the Am-Be source in the laboratory, this spectrum was not used in the simulations.

### Simulations validation with lead

To validate this model, photon mass attenuation coefficients were tested for lead shields for different energies. The MC simulations were tested versus published reference data (33).

### Simulations validation with paraffin

To validate the simulated model for the calculation of neutrons, the macroscopic cross-section for paraffin wax (C_30_H_62_) with a density of 0.88 g/cm^3^ and a monoenergetic neutron source (E _eff_ = 4.5 MeV ^241^Am–Be) was applied using the MC model and compared with the published reference data (34).

### Design of Nanoshield in MCNPX

Nanocomposites were designed as cylinders with a diameter of 30 cm, and the inner space was divided into cubic cells in the nanometer dimension using the lattice feature of MCNPX MC code (Fig. 1b). Nanoshields made of boron carbide and iron oxide NPs 100 nm in diameter in a matrix of silicone resin were simulated (Fig. 1b). These shields were modeled with a weight percentage of 10, 20, 30, 40, and 50 nanomaterials in silicone resin. Shields were simulated with a thickness of 0.5, 1, 1.5, 2, 3, 4, and 5 cm to obtain more accurate macroscopic cross-sections in different densities. Special configurations using two shields were also simulated. This simulation geometry was designed to evaluate the double-layer shielding effect of Fe_3_O_4_ and B_4_C nanoshields on photo-neutrons (Fig. 1c). Two overall thicknesses of 1, 2, 3, and 4 cm (half thickness for each nanoshield) were simulated. Evidently, the lattice represents the silicone resin matrix of shields, and spherical NPs of B_4_C and Fe_3_O_4_ were located at the center of each cube. Depending on the nano-materials and their concentration, and considering the fixed diameter of 100 nm spheres of B_4_C and Fe_3_O_4_, different cube sizes were modeled to obtain different volumes and, consequently, to achieve different concentrations of each nanomaterial in each shield. Initially, nanomaterials were separately used in each shield and tested as a single-layer shield, and subsequently, nano-iron oxide and nano-boron carbide were modeled together as a double-layer shield; the iron oxide shield was placed toward the source and the boron carbide was located toward the detector.

### Fabrication of Nanoshields

**Fig. 3:**
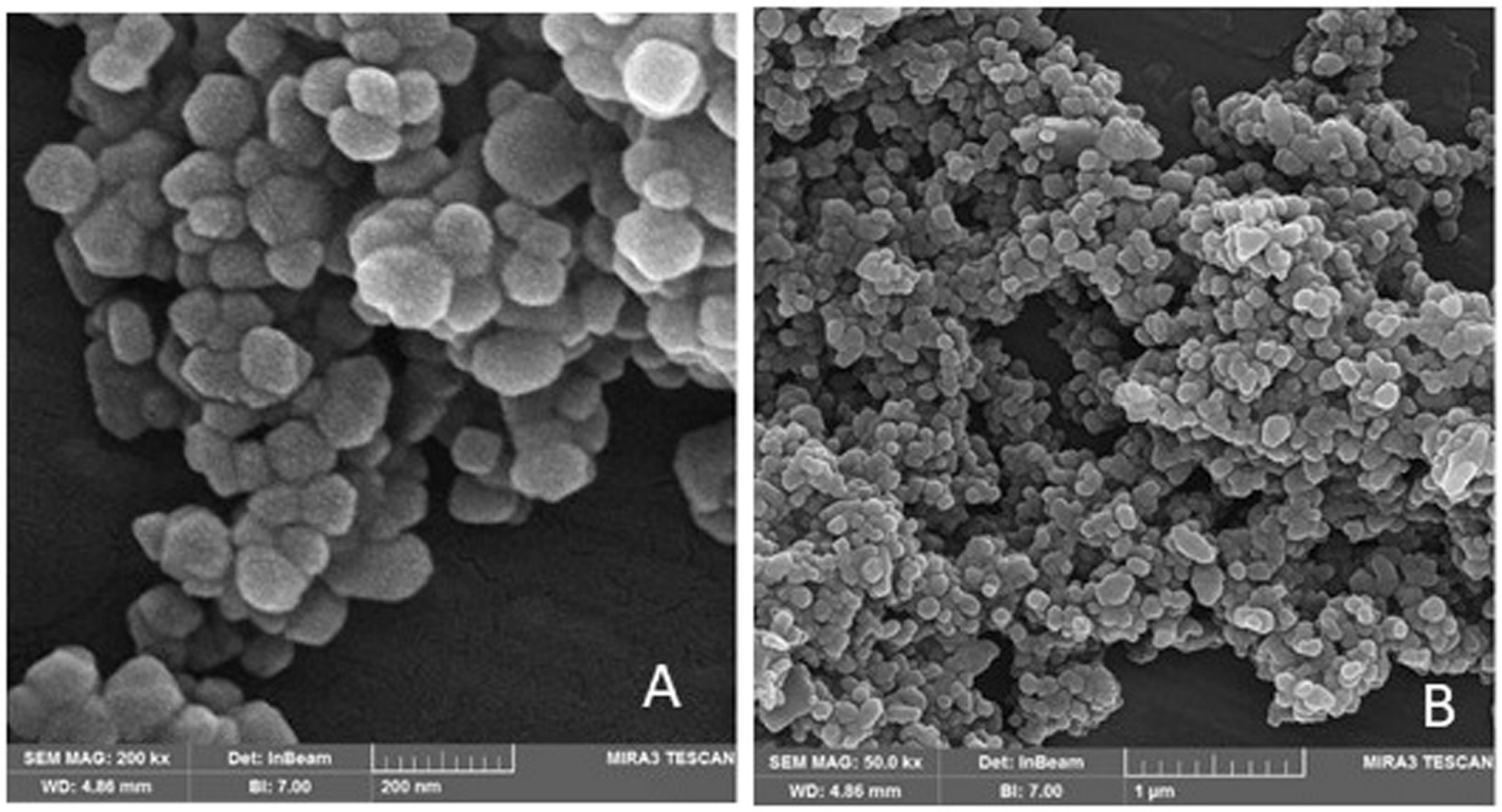
Scanning electron micrograph of NPs from Iranian Nanomaterials Pioneers Company, NANOSANY; iron oxide NPs (**a**), boron carbide NPs (**b**)

In the second step, nanoshields were fabricated based on the results of MC simulation. To fabricate nanoshields, nano-iron oxide and nano-boron carbide were purchased from Iranian Nanomaterials Pioneers Company and the size and homogeneity of NPs were evaluated by scanning electron micrograph (SEM). The diameter of nano-iron oxide and nano-boron carbide was equal to 20–30 nm (Fig. 3a) and 55 nm (Fig. 3b), respectively. Densities of silicone resin, iron oxide, and boron carbide were 1.24, 5.17, and 2.51 g/cm^3^, respectively.

Nanoshields were prepared in cylindrical samples with a diameter of 4 cm and thicknesses of 0.5, 1, 1.5, and 2 cm. They were made by adding iron oxide and boron carbide to the silicone resin. Shields were separately prepared in 10, 20, and 30 wt% (wt.%) of nanomaterial concentration for iron oxide and boron carbide. Four samples of nanoshields were produced for each percentage. Nano-materials were blended with silicone by an electric blender, and a hardener was added to each sample during blending. When NP concentration exceeded 30%, the mixture of silicone resin and nanomaterials did not properly solidify after being mixed.

### Testing the Nanoshields

Fabricated shields were tested in a laboratory with an Am-Be source (Fig. 2c). The source was placed in a water tank with the diameters of 2.5 × 1.5 × 1.5 m (L × W × H) and with 10 cm of pure water between source and shields (the experimental setup is illustrated in Fig. 1d). The BF_3_ neutron detector was covered with cadmium leaves to eliminate scattered neutrons. Shields were placed in front of a neutron detector with a diameter of 2 cm.

Counts were measured in 15-min periods and repeated 3 times for each sample for averaging. The measurements were separately performed for each shield and finally done for the combined shield, including 1 cm of iron oxide close to the source and 1 cm of boron carbide toward the detector side. After the measurement, the macroscopic cross-section for iron oxide and boron carbide shields was calculated using Eq. 1.

## Results

### Experimental measurements and MC calculations uncertainties

The main sources of experimental uncertainties included limited accuracy of the BF3 detector, uncontrolled changes to the environment and conditions (e.g., receiving the scatted neutrons to the detector), limitations, and simplifications of the experimental procedure. In this regard, the uncertainties from the experimental setup and repeated measurements were negligible and are provided in the related sections. It is noteworthy that the statistical uncertainties of the results from the MC simulations were less than 1% in all cases.

### Simulations validation with lead

**Fig. 4:**
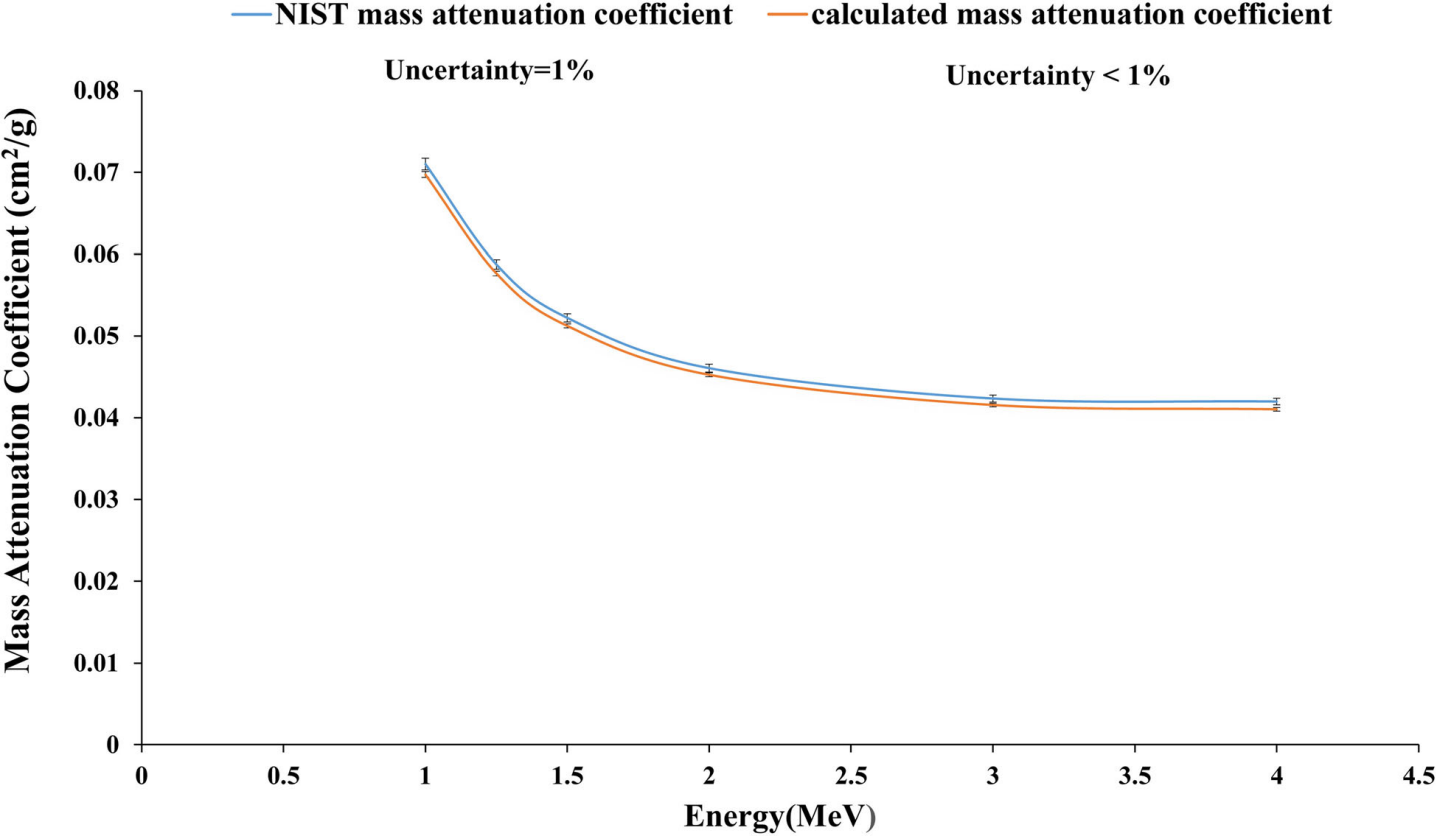
Comparison between Mass attenuation coefficients of lead obtained from NIST and simulation geometry for simulation validation

The results of the simulation for the photon source and lead shield were compared with the data from the National Institute of Standards and Technology (NIST) [33]. These results demonstrated a difference of < 1.8% with data of lead attenuation from NIST (Fig. 4).

### Simulations validation with paraffin

For neutrons’ source and paraffin wax, simulation results were compared with experimental data [34]. The values of the macroscopic cross-section for experimental [34] and MC methods were 0.192 ± 0.014 and 0.195 cm^1^, respectively. Considering uncertainties in neutron calculations (less than 0.01), the resulting difference of 1.5% was acceptable for our MC model validation.

### Macroscopic cross-section of Nanoshields for Monoenergetic neutrons calculated by the MC method

**Fig. 5:**
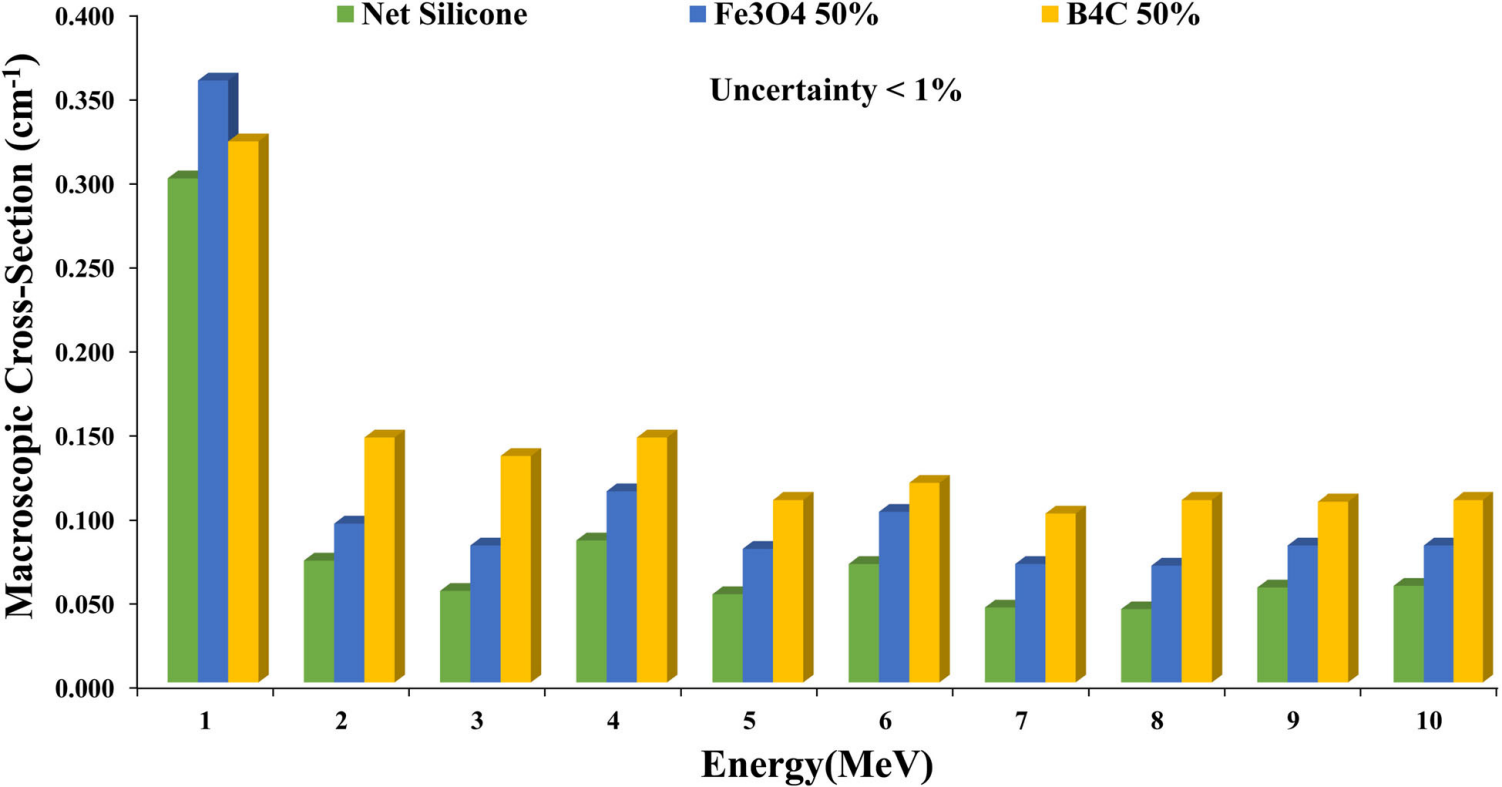
Comparison of the macroscopic cross-sections of iron oxide and boron carbide nanoshields (50% NPs) and net silicone for discrete neutron energies (1 to 10 MeV), extracted from neutron fluence attenuation for shields with thicknesses of 0.5, 1, 1.5, 2, 3, 4, and 5 cm

The macroscopic cross-sections of boron carbide and iron oxide nanocomposites were calculated for several weight percentages (wt.%) of NPs and 10 consecutive energies. For simplicity, only the results of composites with a concentration of 50 wt.% are presented in Fig. 5. In general, attenuation efficiency decreases with the increase in energy, but it shows some fluctuations. In addition, attenuation efficiency is significantly higher (approximately double) for both boron carbide and iron oxide at 1 MeV compared to higher energies (2 to 10 MeV). In all concentrations of NPs, the macroscopic cross-section was higher for boron carbide relative to iron oxide in the energy range of 2–10, but for 1 MeV, iron oxide acted superior to boron carbide. In other words, the macroscopic cross-section of iron oxide was ~ 11% higher than that of boron carbide in 50 wt.% concentration.

### Macroscopic cross-section of Nanoshields for neutron Spectrum of am-be source calculated by the MC method

**Fig. 6:**
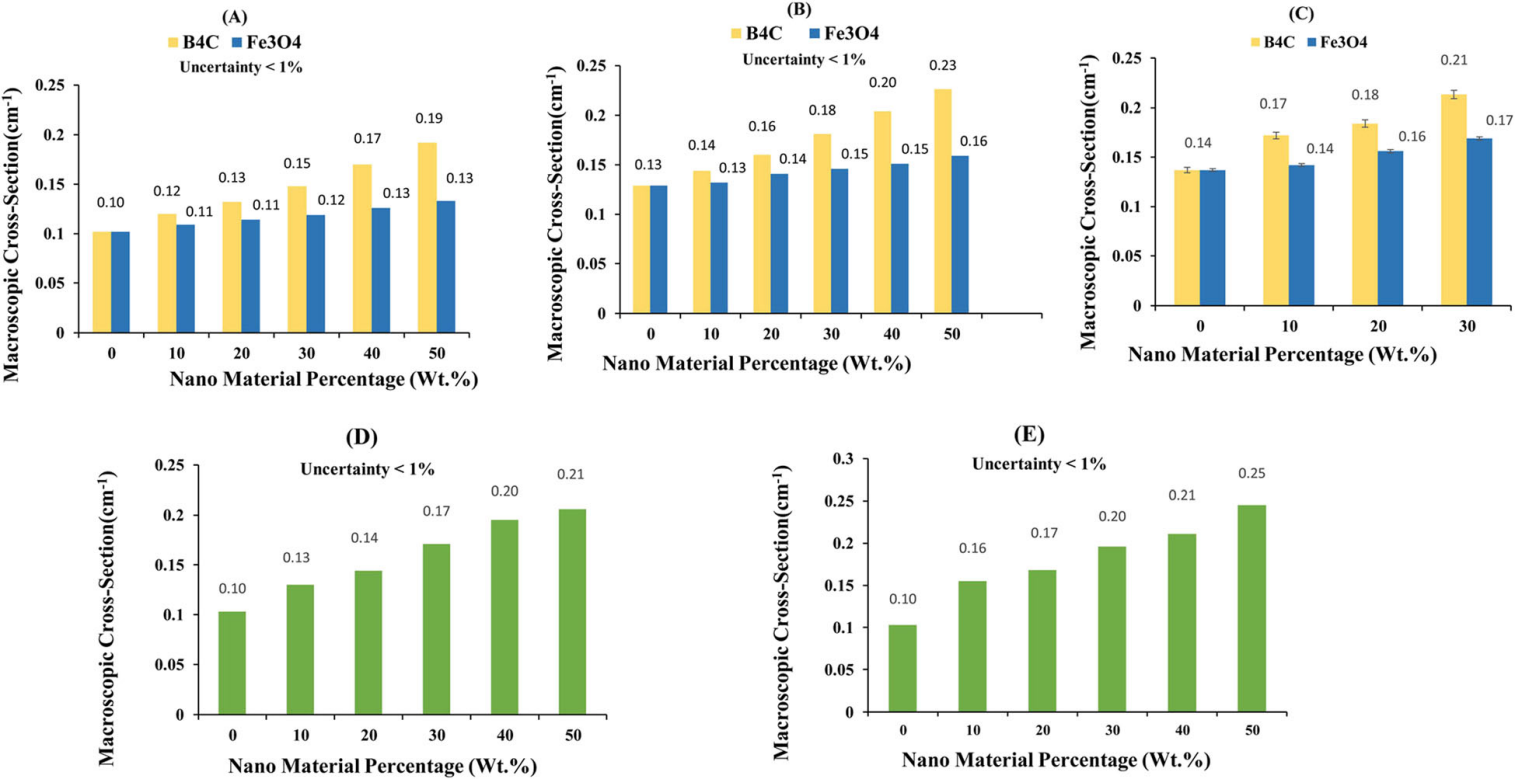
Comparison of simulation and measurement results of the macroscopic cross-sections of iron oxide and boron carbide single and double-layer nanoshields: single-layer shields in front of Am-Be source-MC (**a**), single-layer shield in front of linac spectrum-MC (**b**), single-layer shield in front of Am-Be source in the laboratory (**c**), double-layer shields in front of Am-be source-MC (**d**), double-layer shield in front of linac source-MC (**e**)

The applied neutron spectrum (Fig. 2a) had an energy range between 0.4 eV and ~ 15 MeV, and mainly from 1 to 3 MeV. Transmission factors for mono- and double-layer nanocomposites in front of the Am-Be source were calculated by the MC method (Tables 1 and 2). Based on Table 1, B_4_C had a superior ability in eliminating neutrons compared to Fe_3_O_4_ for all concentrations, and this superiority increased with filler concentration. At the concentration of 10 wt.%, both nanocomposites showed similar attenuation, but with an increase in NP concentration, boron carbide exhibited more efficiency in eliminating neutrons. The macroscopic cross-sections of nanocomposites are depicted in Fig. 6a, which demonstrates the difference between attenuation yields of two nanocomposites. For instance, the macroscopic cross-section for boron carbide rose from 0.12 cm^−1^ for 10% to 0.192 cm^−1^ for 50%. However, for iron oxide, it was 0.109 cm^−1^ for 10 wt.% and increased to 0.133 cm^−1^ for 50 wt.%. Furthermore, the cross-section for the double-layer shield was equal to 0.21 cm^−1^ for the concentration of 50% (Fig. 6d).

**Table 1:** Transmission factor comparison for mono-layer nanomaterial shields for Am-Be and linac spectra simulation (Uncertainties < 0.01)

Nano Material Concentration	Samples Thickness (cm)	Am-Be Spectrum (I/I_0_)		Linac Spectrum (I/I_0_)	
**Net Silicone**	0.5	0.94		0.94	
	1	0.91		0.87	
	1.5	0.85		0.85	
	2	0.82		0.76	
	3	0.74		0.67	
	4	0.67		0.59	
	5	0.60		0.52	
	**Samples Thickness (cm)**	**Am-Be Spectrum (I/I**_**0**_**)**		**Linac Spectrum (I/I**_**0**_**)**	
		**Fe**_**3**_**O**_**4**_	**B**_**4**_**C**	**Fe**_**3**_**O**_**4**_	**B**_**4**_**C**
**10% Wt. nano material, 90%Wt. silicone**	0.5	0.95	0.94	0.93	0.93
	1	0.89	0.88	0.87	0.85
	1.5	0.85	0.83	0.82	0.80
	2	0.79	0.79	0.75	0.73
	3	0.71	0.69	0.66	0.63
	4	0.64	0.62	0.59	0.55
	5	0.58	0.55	0.51	0.49
**20% Wt. nano material, 80%Wt. silicone**	0.5	0.95	0.94	0.93	0.92
	1	0.88	0.88	0.87	0.84
	1.5	0.84	0.81	0.81	0.78
	2	0.79	0.76	0.75	0.72
	3	0.71	0.67	0.65	0.61
	4	0.63	0.59	0.57	0.53
	5	0.57	0.52	0.49	0.45
**30% Wt. nano material, 70%Wt. silicone**	0.5	0.94	0.93	0.93	0.92
	1	0.88	0.86	0.86	0.83
	1.5	0.83	0.80	0.79	0.76
	2	0.79	0.74	0.74	0.69
	3	0.70	0.64	0.64	0.58
	4	0.62	0.55	0.55	0.48
	5	0.55	0.48	0.49	0.40
**40% Wt. nano material, 60%Wt. silicone**	0.5	0.94	0.92	0.93	0.90
	1	0.87	0.84	0.85	0.81
	1.5	0.82	0.77	0.79	0.73
	2	0.77	0.71	0.73	0.66
	3	0.68	0.60	0.63	0.54
	4	0.60	0.50	0.54	0.44
	5	0.53	0.43	0.47	0.36
**50% Wt. nano material, 50%Wt. silicone**	0.5	0.94	0.91	0.93	0.89
	1	0.87	0.82	0.84	0.79
	1.5	0.81	0.75	0.77	0.71
	2	0.76	0.68	0.72	0.63
	3	0.67	0.57	0.62	0.51
	4	0.59	0.45	0.53	0.40
	5	0.51	0.39	0.45	0.33

**Table 2:** Transmission factor comparison for double-layer nanoshields for Am-Be source and linac spectra simulation (Uncertainties < 0.01)

Nano Material Concentration	Samples thickness (cm)	Am-Be Spectrum (I/I_0_)	Linac Spectrum (I/I_0_)
**Net silicone**	1	0.91	0.87
	2	0.82	0.76
	3	0.74	0.67
	4	0.67	0.59
**10% Wt. nano material, 90%Wt. silicone**	1	0.88	0.85
	2	0.77	0.73
	3	0.68	0.62
	4	0.59	0.54
**20% Wt. nano material, 80%Wt. silicone**	1	0.86	0.84
	2	0.76	0.71
	3	0.65	0.60
	4	0.56	0.51
**30% Wt. nano material, 70%Wt. silicone**	1	0.84	0.83
	2	0.73	0.67
	3	0.61	0.55
	4	0.50	0.46
**40% Wt. nano material, 60%Wt. silicone**	1	0.83	0.80
	2	0.70	0.63
	3	0.58	0.52
	4	0.45	0.43
**50% Wt. nano material, 50%Wt. silicone**	1	0.81	0.77
	2	0.67	0.60
	3	0.55	0.49
	4	0.44	0.37

The transmission factors for the concentration of 50% and a thickness of 4 cm were 0.59 and 0.45 for mono-layer shields of iron oxide and boron carbide, respectively, and 0.44 for the double-layer shield (Tables 1 and 2).

### Macroscopic cross-section of Nanoshields for neutron Spectrum of am-be source determined by experiment

The Am-Be source in the laboratory has an energy peak at 2.31 MeV (Fig. 2c). The spectrum was provided by the company after installing the Am-Be source. Table 3 shows the experimentally measured transmission factor in different thicknesses and for various percentages of NPs. With an increase in nanomaterial concentration, boron carbide shows more efficiency in eliminating neutrons. At the percentage of 30 and a thickness of 2 cm, it reduces the number of neutrons by 34%, compared to 29% for iron oxide. As expected, attenuation for the double-layer shield is more than two nanoshields separately. The transmission factor for the double-layer shield with a thickness of 2 cm is 0.62 (Table 3).

**Table 3:** Transmission factor comparison for experimental nanoshields with Am-Be source

Nano Material Concentration	Samples thickness (cm)	Net silicone (I/I_0_)	
**Net silicone**	0.5	0.96 ± 0.002	
	1	0.90 ± 0.003	
	1.5	0.84 ± 0.005	
	2	0.76 ± 0.009	
	**Samples thickness (cm)**	**Fe**_**3**_**O**_**4**_**(I/I**_**0**_**)**	**B**_**4**_**C (I/I**_**0**_**)**
**10% Wt. nano material, 90%Wt. silicone**	0.5	0.95 ± 0.002	0.95 ± 0.003
	1	0.89 ± 0.002	0.86 ± 0.01
	1.5	0.82 ± 0.006	0.79 ± 0.01
	2	0.75 ± 0.007	0.71 ± 0.007
**20% Wt. nano material, 80%Wt. silicone**	0.5	0.95 ± 0.002	0.92 ± 0.007
	1	0.87 ± 0.007	0.83 ± 0.005
	1.5	0.80 ± 0.009	0.75 ± 0.001
	2	0.74 ± 0.003	0.70 ± 0.01
**30% Wt. nano material, 70%Wt. silicone**	0.5	0.92 ± 0.006	0.89 ± 0.006
	1	0.85 ± 0.01	0.79 ± 0.005
	1.5	0.78 ± 0.003	0.70 ± 0.004
	2	0.71 ± 0.01	0.66 ± 0.02
**Double layer (Fe**_**3**_**O**_**4**_**and B**_**4**_**C)**	**2**	**I/I**_**0**_ **= 0.62 ± 0.01**	

The macroscopic cross-section in Fig. 6c demonstrates the difference between attenuation yields of the mentioned nanomaterials. The macroscopic cross-section for boron carbide rises by 0.04 cm^− 1^ from 10 to 30%, while this increase is 0.03 cm^− 1^ for iron oxide.

The experimental results for monolayer shields of Fe3O4 and B_4_C and double-layer shields (Table 3) demonstrate satisfactory agreement with the simulation results (Tables 1 and 2). The difference between MC and measurements is in the range of 10%. There was about 9% difference between experimental measurement of neutron attenuation and MC results, which can be attributed to the discrepancy between fabricated nanocomposites where the NPs were heterogeneously distributed inside the resin matrix. However, in the MC simulations, NPs were homogenously dispersed with the resin matrix. Besides, the uncertainties in providing energy spectra during the installation of the Am-Be neutron source as experimentally and subsequently measurements (about 5%), primarily due to the uncertainty in the source strength, had a non-negligible effect on the observed discrepancy between MC and measurement results.

### Macroscopic cross-section of Nanoshields for a neutron Spectrum from Linac calculated by the MC method

The applied neutron spectrum of linac (Fig. 2a) has an energy range between 40 keV and ~ 10 MeV and contains a peak at 1 MeV. Tables 1 and 2 present the attenuation in different thicknesses and for various percentages of NPs for mono- and double-layer nanoshields. As can be seen, attenuation is higher for the double-layer shield than mono-layer shields. Based on the comparison of transmission factor for mono-layer and double-layer shields (concentration of 50% and thickness of 4 cm), the values for iron oxide, boron carbide, and double-layer shields were 0.53, 0.40, and 0.37, respectively. Attenuation increases for both nanocomposites by an increase in NP concentration (wt.%), and at 10 wt.%, both nanocomposites show similar attenuation. However, with an increase in the weight percentage of boron carbide, it shows more efficiency in eliminating neutrons. At the percentage of 50 and a thickness of 5 cm, it reduces the number of neutrons by 67% (Table 1). The reduction is only 55% for iron oxide (Table 1).

The macroscopic cross-section in Fig. 6b indicates the difference between attenuation yields of two nanocomposites. The macroscopic cross-section for boron carbide rose from 0.144 cm^−1^ for 10 wt.% to 0.226 cm^−1^ for 50 wt.%. For iron oxide, it was 0.132 cm^−1^ for 10 wt.% and increased to 0.159 cm^−1^ for 50 wt.%. According to Fig. 6e, for double-layer shields the macroscopic cross-section increased from 0.16 cm^−1^ for the concentration of 10% to 0.25 cm^−1^ for 50 wt.%. It is of note that for each spectra, a permanent increase is observed in attenuation as the concentration of the NPs increases (Fig. 6).

**Fig. 7:**
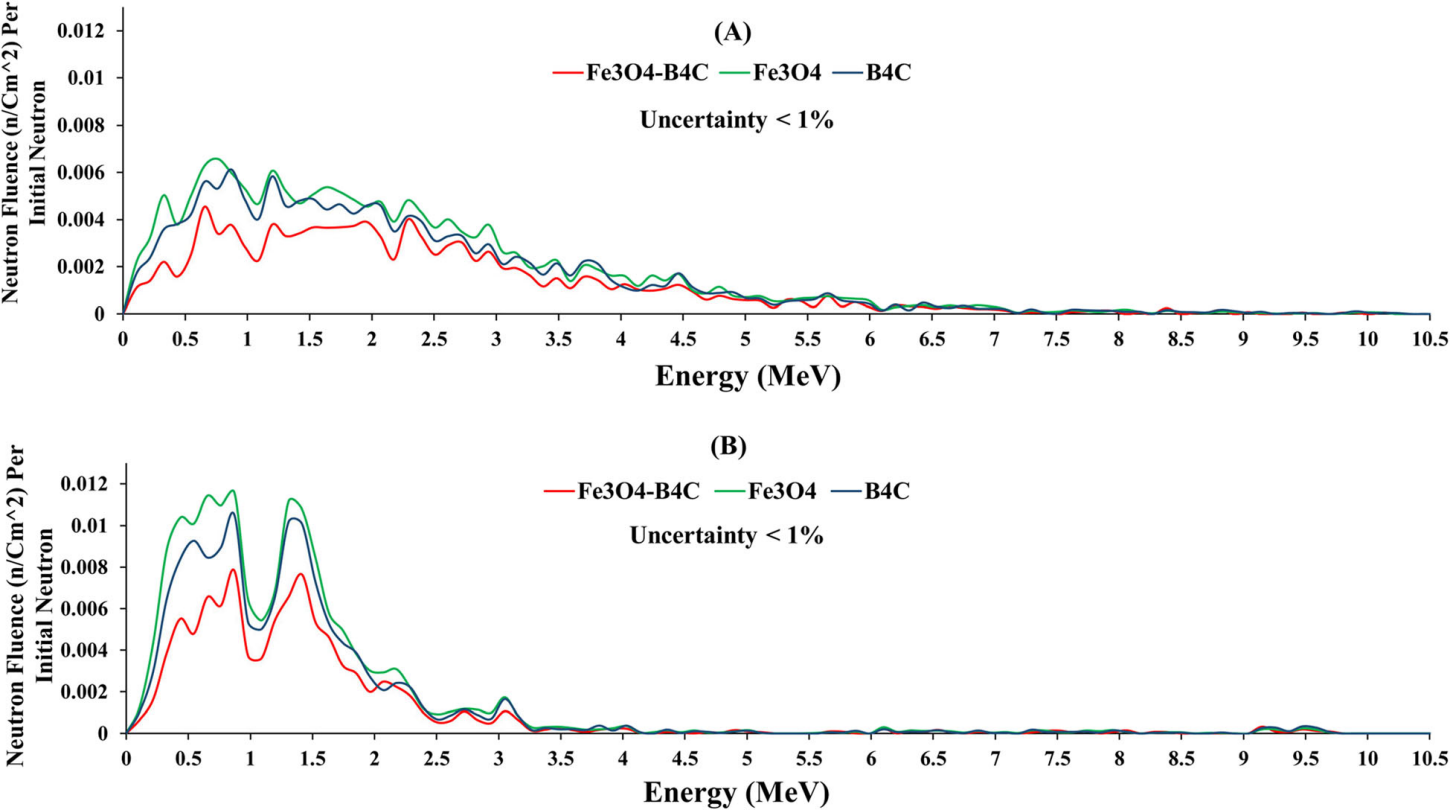
Simulated energy spectra for neutrons reaching the detector passing through a single and double-layer of nanoshields (50% NPs): Am-Be source (**a**), linac spectrum (**b**)

Figure 7 a and b demonstrate the energy spectra of neutrons reaching the detector for a total thickness of 2 cm of all studied nanocomposites (50% NPs) in front of the Am-Be and linac spectra, respectively. These nanocomposites include 2 cm of iron oxide, boron carbide, and 2 cm of double-layer shields (1 cm for iron oxide and 1 cm for boron carbide).

## Discussion

Studies have shown the production of neutrons during radiation therapy with high-energy photons (E > 10 MV) [2, 3, 35]. To eliminate these neutrons, shields such as concrete, B_4_C, and other materials have been designed to efficiently remove thermal neutrons [6, 21, 36]. Other materials such as Fe_3_O_4_ are utilized for shielding fast neutrons [17, 26]. Recent investigations have reported the advantage and higher efficiency of composite shields with fine and nano-sized particles over larger or micro-sized particles as neutron shields [21–23]. In this study, silicone resin was chosen as the shield matrix because of its better flexibility, biocompatibility, and durability against humidity and physical damage [37]. Considering the macroscopic cross-section of net silicone resin in all simulation surveys, because of its low atomic weight (which increases the probability of elastic interaction between neutron and nucleus), it shows acceptable neutron attenuation and can be considered as an effective part of the shield. In the present research, neutron attenuation was assessed in silicone resin loaded with different concentrations of nano-sized boron carbide and iron oxide in three simulated spectra and one experimental spectrum of Am-Be source.

In the MC simulation, monoenergetic neutrons were applied during the calculations to provide more information concerning the attenuation efficiencies of samples for different neutron energies. A comparison of both types of nanoshields showed that neither had complete superiority over the other in the entire range of neutron energies (Table 1). Examination of the attenuation in each monoenergetic neutron source revealed that each nanocomposite had its own merits in specific neutron energy (Fig. 5). It can be claimed that both Fe_3_O_4_ and B_4_C have the highest neutron attenuation in 1 MeV energy (Fig. 5), which corresponds to the peak of the linac energy spectrum (Fig. 2a). Moreover, the previous simulation and experimental results indicated that the contribution of thermal neutrons on the neutron spectra from the modern linacs is slight and about 6% [38, 39]. Thus, a shield consisting of Fe_3_O_4_ and B_4_C can be more effective in shielding patients against neutrons produced by linac head during radiotherapy.

For MC simulation (Am-Be and linac spectra) and measurement with Am-Be source, both shields demonstrated acceptable efficiency in attenuating neutrons, but boron carbide was superior in eliminating neutrons compared with iron oxide (Fig. 6a, b, and c and Table 1). As expected, the simulated double-layer shield exhibited greater macroscopic cross-section relative to both monolayer Fe_3_O_4_ and B_4_C nanocomposites (Figs. 6d and e; Tables 1 and 2). The efficiency of nanocomposites in eliminating neutrons from the linac spectrum was similar to the results of the Am-Be spectrum in the MC simulations. Nevertheless, it showed a slightly higher neutron macroscopic cross-section (Figs. 6d and e; Tables 1 and 2), which can be attributed to the lower neutron energy of the linac spectrum. Besides, the discrepancy between two nanocomposites in terms of the macroscopic cross-section was higher for the linac spectrum (Fig. 6b). Similar to other neutron spectra studied in our investigation, the double-layer shield showed a larger macroscopic cross-section compared to other monolayer nanocomposites (Tables 1 and 2).

It can be deducted from Fig. 7a (detected spectra) that neutron energy spectra of Am-Be source have attenuated and shifted to lower energies relative to initial spectra (Fig. 2a) and minimum attenuation occurred in energy regions between 0.5–1 MeV for both nano materials. Moreover, at the energy of less than 3 MeV, boron carbide acts better than iron oxide. The neutron energy spectra from the linac source (Fig. 7b) also declined but with fluctuations without any shifting to lower energies relative to initial spectra (Fig. 2a). The maximum reduction occurred at the energy peak of initial spectra (~ 1 MeV) for both nano materials. In addition, in lower energy regions, boron carbide acts better than iron oxide, but the double-layer shield has the highest attenuation for both sources.

In the current study, the weights of experimentally made shields with thickness and radius of 2 cm for net silicone, iron oxide (50% concentration), and boron carbide (50% concentration) were 31, 60, and 41 g, respectively, and for the double-layer shield, it was 50.5 g. It should be noted that the matrix of silicone resin produces an additional shielding for neutrons due to its low atomic weight. In addition, its mechanical properties make the shield suitable for application as a flexible sheet on the patient. According to our calculations and considering the weight of the nanocomposite, a double-layer nanocomposite can be used as a flexible and light shield on the patient body. For instance, a 2-cm-thick double-layer nanocomposite (50% concentration) will result in a 40% reduction in the patient’s received neutron fluence during radiation therapy with the 18 MeV photon beam (Table 2). It is of note that the reduction in the neutron fluence increases with nanoshield thickness and concentration of NPs so that a double-layer of nanocomposite with 4 cm in thickness and 50% concentration can reduce the neutron fluence for 18 MeV photon beam of the linac by 63% (Table 2).

Several studies have been conducted to evaluate the neutron cross-section of different materials and compounds. For instance, D’Mellow et al. and Soltani et al. reported a high efficiency of boron carbide in removing thermal neutrons [21, 36]. Also, El-Khayatt et al. and Tellili et al. assessed different materials for shielding fast neutrons. Among these materials, iron oxide has an acceptable cross-section against fast neutrons [40, 41]. Nevertheless, before the present study, there was no study on the combined effect of double-layer nanocomposites in eliminating photo-neutrons from the linac. In this context, evaluating the other nanoshields to eliminate thermal and fast neutrons simultaneously from sources with many thermal and fast neutrons may be a topic for future investigations.

## Conclusion

According to the results of this study, iron oxide-doped nanocomposite acted better in eliminating and attenuating fast neutrons, mostly around 1 MeV relative to boron carbide-doped nanocomposite. Our designed double-layer nanocomposite showed greater efficiency in eliminating fast neutrons. The results of MC simulations and the experimental test thoroughly comply with the mentioned statement. Considering the low weight and flexibility of the designed double-layer nanocomposite, it can be efficiently applied for protecting patients against neutrons produced from the linac head during radiation therapy with the 18 MeV photon beam.

## Data Availability

The dataset used and analyzed during the current study are available from the corresponding author on reasonable request.

## Abbreviations

Linac: Medical linear accelerator
NPs: Nanoparticles
MC: Monte Carlo
RBE: Relative biological effectiveness
SEM: Scanning electron micrograph
NIST: National institute of standards and technology
